# An Artificial Peptide-Based Bifunctional HIV-1 Entry Inhibitor That Interferes with Viral Glycoprotein-41 Six-Helix Bundle Formation and Antagonizes CCR5 on the Host Cell Membrane

**DOI:** 10.3390/v15051038

**Published:** 2023-04-23

**Authors:** Chao Wang, Qing Li, Lujia Sun, Xinling Wang, Huan Wang, Wenpeng Zhang, Jiahui Li, Yang Liu, Lu Lu, Shibo Jiang

**Affiliations:** 1 State Key Laboratory of Toxicology and Medical Countermeasures, Beijing Institute of Pharmacology and Toxicology, 27 Tai-Ping Road, Beijing 100850, China; 2 Key Laboratory of Medical Molecular Virology (MOE/NHC/CAMS), School of Basic Medical Sciences and Shanghai Public Health Clinical Center, Shanghai Institute of Infectious Diseases and Biosecurity, Fudan University, 131 Dong An Road, Shanghai 200032, China; 3 Key Laboratory of Structure-Based Drug Design and Discovery of the Ministry of Education, Shenyang Pharmaceutical University, Shenyang 110016, China

**Keywords:** HIV-1, gp41, CCR5, entry inhibitors, multitarget-directed ligands, coiled coil

## Abstract

Human immunodeficiency virus type 1 (HIV-1) is characterized by high variability and drug resistance. This has necessitated the development of antivirals with a new chemotype and therapy. We previously identified an artificial peptide with non-native protein sequence, AP3, with the potential to inhibit HIV-1 fusion through targeting hydrophobic grooves on the N-terminal heptad repeat trimer of viral glycoprotein gp41. Here, a small-molecule HIV-1 inhibitor targeting chemokine coreceptor CCR5 on the host cell was integrated into the AP3 peptide, producing a novel dual-target inhibitor with improved activity against multiple HIV-1 strains including those resistant to the currently used anti-HIV-1 drug enfuvirtide. Its superior antiviral potency in comparison with the respective pharmacophoric moieties is in consonance with the dual binding of viral gp41 and host factor CCR5. Therefore, our work provides a potent artificial peptide-based bifunctional HIV-1 entry inhibitor and highlights the multitarget-directed ligands approach in the development of novel therapeutic anti-HIV-1 agents.

## 1. Introduction

The alarming rate at which human immunodeficiency virus type 1 (HIV-1) is developing resistance to current antiretroviral therapy (ART) represents one of the major global challenges to the treatment of HIV-1/AIDS patients [1]. This requires the constant search for novel anti-HIV-1 drugs that affect different stages of the viral life cycle [2]. Up to now, more than 30 antiretroviral drugs have been licensed by the U.S. FDA for clinical use [3]. Among them, HIV-1 entry inhibitors acting on the virus–cell fusion stage combat HIV-1 outside host cells to prevent the integration of the viral genome into the host genome, thus providing an efficient therapeutic or prevention strategy for blocking HIV-1 infection [4,5,6].

**Figure 1:**
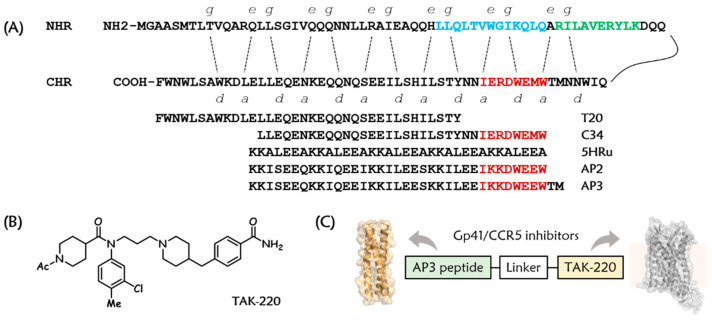
HIV-1 gp41 NHR-CHR interaction and entry inhibitors. (**A**) Schematic view of interactions between the NHR and CHR. Residues at the *a* and *d* positions in the CHR interact with *e* and *g* residues in the NHR, respectively. Sequences corresponding to the NHR pocket-1 (blue), pocket-2 (green), and the CHR pocket-binding domain (PBD) (red) are respectively marked. (**B**) Structure of the small-molecule CCR5 antagonist TAK-220. (**C**) Targets for the development of bifunctional inhibitors. The HIV-1 gp41 NHR trimer (orange, adapted from PDB ID: 1AIK) and CCR5 (gray, adapted from PDB ID: 4MBS) are shown as cartoons together with the schematic representation of AP3-linker-TAK-220 chimera.

Entry of HIV-1 into target cells is mediated by its envelope glycoprotein (Env) surface subunit gp120 and transmembrane subunit gp41 [7,8]. After gp120 binding to the cellular CD4 receptor and coreceptor, either C-C chemokine receptor 5 (CCR5) or C-X-C chemokine receptor 4 (CXCR4), the exposed N-terminal heptad repeat (NHR) and C-terminal heptad repeat (CHR) regions of gp41 form a six-helix bundle (6-HB) coiled-coil structure that brings viral and host cell membranes into close proximity, thus facilitating their fusion. However, during gp41-mediated HIV-1 infection, the NHR trimers become transiently exposed and thus accessible to fusion inhibitory peptides derived from the CHR (Figure 1A) [9,10,11]. T20 (brand name: Fuzeon; generic name: enfuvirtide) is currently the only U.S. FDA-approved HIV-1 fusion inhibitor [12,13]. However, the T20 peptide requires high dosages (90 mg twice a day) due to its relatively low efficacy and short in vivo half-life. Furthermore, T20 easily induces drug resistance that confounds treatment and has led to a growing number of patients for whom T20 is ineffective [11]. In addition, through direct competition to the NHR site, or by sequestering/binding T20 to form T20–antibody complexes, the preexisting antibodies in HIV-1-infected patients could interfere with the natural peptide T20-mediated inhibition of membrane fusion [14]. These drawbacks call for new strategies in the development of next-generation peptide fusion inhibitors that are able to intercept the virus before it invades the target cells.

To address these issues, de novo designed α-helical peptides nonhomologous with the naturally occurring gp41 protein sequence have been created. These peptides mimic the secondary structural features of CHR motifs within the HIV-1 6-HB and act as antiviral agents that block viral fusion. Over the past decade, our group has developed a series of novel HIV-1 fusion inhibitors based on artificial peptide sequences. For instance, after systematic optimization of the universal heptad repeat sequence-based α-helical peptide 5HR_u_, the engineered AP2 showed significantly improved antiviral activity [15,16,17,18]. Based on the sequence of AP2, a more potent AP3 peptide, which inhibits the infection of divergent HIV-1 isolates including those resistant to T20, was designed by adopting an M-T hook strategy (Figure 1A) [19]. This promising hit prompted us to focus on the optimization of AP3 to develop an artificial α-helical peptide possessing even higher anti-HIV-1 activity.

Very recently, we discovered a multitarget-directed ligands (MTDLs) strategy for designing bifunctional HIV-1 entry inhibitors able to simultaneously modulate multiple interdependent targets within the highly cooperative stages of HIV-1 entry [20]. A CCR5 antagonist-based HIV-1 entry inhibitor, TAK-220 (Figure 1B), was covalently linked to the carboxy terminus of a CHR-based peptide, C34, widely used as a fusion inhibitor design template [21]. Consequently, a dual-target molecule was identified, leading to a potent HIV-1 entry inhibitor with markedly improved antiviral potency when compared to either of the pharmacophores alone. Considering the above findings, we reasoned that the MTDLs concept could be applied to bifunctional artificial α-helical peptides with improved anti-HIV-1 efficacy. Accordingly, in this study, we utilized a small-molecule CCR5 antagonist to modify a de novo designed HIV-1-neutralizing peptide and observed a significant optimization (Figure 1C). Significantly, the resulting peptide AP3P4E had highly enhanced potency over that of the currently used T20 and could inhibit a variety of HIV-1 isolates. These results suggest that AP3P4E is an ideal candidate for further development in clinical use. Moreover, this multifunctional agent represents a promising advancement in the design of artificial anti-HIV-1 therapeutics.

## 2. Materials and Methods

### 2.1. Chemistry

Unless otherwise stated, all materials were obtained from commercial sources and used without further purification. Thin-layer chromatography (TLC) was performed using GF254 silica gel plates and column chromatography was performed using 200–300 mesh silica gel from Qingdao Haiyang Chemical Co. (Qingdao, China). Electrospray ionization mass spectroscopy was carried out with an API150 mass spectrometer from ABI Inc. (Foster City, CA, USA). ^1^H NMR and ^13^C NMR spectra were obtained in a JNM-ECA-400 spectrometer in chloroform-*d*, acetone-*d6*, or DMSO-*d6* operating at 400 MHz (^1^H NMR) and 100 MHz (^13^C NMR). Chemical shifts were reported as δ values (ppm) relative to TMS δ = 0 (^1^H) as the internal standard (IS), and coupling constants (*J*) were reported in Hz. Multiplicities were recorded as s (singlet), d (doublet), t (triplet), q (quartet), dd (doublet of doublets), dt (doublet of triplets), and m (multiplet). Final purity was determined by analytical reversed-phase high-performance liquid chromatography (RP-HPLC) analysis on a Shimadzu analytical HPLC system using a Waters Bridge C8 column (250 mm × 4.6 mm, 5 µm), linear gradient elution with H_2_O/ACN containing 0.1% TFA, and UV detection at 210 nm (Tables S1 and S2). The purity of each tested compound was confirmed to be ≥95% by analytical RP-HPLC. Such information is provided in the Supplementary Materials. The molecular weight of the peptides was confirmed by MALDI-TOF-MS (Autoflex III, Bruker Daltonics, Billerica, MA, USA).

Synthesis of Compound **2**. A mixture of acetic anhydride (20 mL, 0.21 mol) and formic acid (10 mL, 0.25 mol) was stirred at 60 °C for 2 h, then cooled to 0 °C. To the solution was added 3-chloro-4-methylaniline **1** (15 g, 0.11 mol) dropwise, and the mixture was stirred at room temperature for about 18 h. After dilution with ethyl ether (Et_2_O, 50 mL) and ethyl acetate (EtOAc, 25 mL), the mixture was washed with water (2 × 50 mL) followed by 1 N aqueous NaOH (3 × 50 mL) and brine (3 × 50 mL), dried over MgSO_4_. The filtrate was concentrated in vacuo, then the residue was triturated with EtOAc/i-PrOH, collected by filtration, and dried in vacuo to afford compound **2** (15.55 g, 85.96%) as a white solid. ^1^H NMR (400 MHz, DMSO-*d6*) δ 10.27 (s, 1H), 8.77 (dd, *J* = 11.3, 4.0 Hz, 1H), 8.30–8.23 (m, 1H), 7.78 (d, *J* = 2.7 Hz, 1H), 7.39–7.33 (m, 1H), 2.25 (s, 3H). ^13^C NMR (100 MHz, DMSO-*d6*) δ 159.74, 137.36, 133.09, 131.28, 130.28, 119.20, 117.75, 18.94. MS calcd for C_8_H_8_ClNO, 169. ESI-MS: 170.0 (M + H)^+^.

Synthesis of Compound **3**. To a solution of 1-bromo-3-chloropropane (15 g, 0.11 mol) in 80 mL of acetone were added **2** (15.55 g, 0.092 mol) and Cs_2_CO_3_ (31.07 g, 0.095 mol), and the mixture was stirred at 100 °C for 8 h. After cooling to room temperature, the mixture was filtered, and the filtrate was concentrated in a vacuum. The residue was diluted with ethyl acetate (EtOAc, 100 mL), washed with water (2 × 100 mL) and brine (2 × 100 mL), dried over MgSO_4_, filtered, and concentrated in vacuo. The residue was purified by column chromatography (EtOAc/MeOH 50:1 to 30:1) to give 18.00 g (79.86%) of **3** as a pale-yellow oil. ^1^H NMR (400 MHz, DMSO-*d6*) δ 8.41 (s, 1H), 7.53 (dd, *J* = 7.7, 2.2 Hz, 1H), 7.44–7.36 (m, 1H), 7.28 (dd, *J* = 8.2, 2.3 Hz, 1H), 3.95–3.84 (m, 2H), 3.62 (q, *J* = 7.1, 6.5 Hz, 2H), 2.33 (s, 3H), 1.95–1.81 (m, 2H). ^13^C NMR (100 MHz, DMSO-*d6*) δ 162.19, 139.73, 133.85, 133.42, 131.82, 123.62, 122.07, 42.56, 41.12, 30.16, 18.97. MS calcd for C_11_H_13_Cl_2_NO, 245. ESI-MS: 246.1 (M + H)^+^.

Synthesis of Compound **4**. To a stirred solution of **3** (18 g, 0.073 mol) in i-PrOH (200 mL) was added concentrated HCl (18 mL), and the mixture was stirred at 60 °C for 3 h, then cooled to room temperature. The mixture was concentrated in vacuo, washed with i-PrOH (2 × 50 mL), and filtered. Then, the filtrate was concentrated in vacuo and dried to afford **4** (18.2 g, 78.82%) as a white solid. ^1^H NMR (400 MHz, chloroform-*d*) δ 7.31 (d, *J* = 2.2 Hz, 1H), 7.25 (s, 1H), 7.23 (s, 1H), 7.13 (dd, *J* = 8.2, 2.3 Hz, 1H), 3.57 (t, *J* = 6.1 Hz, 2H), 3.43–3.34 (m, 2H), 2.35 (s, 3H), 2.22–2.10 (m, 2H). ^13^C NMR (100 MHz, chloroform-*d*) δ 136.50, 136.01, 135.82, 132.30, 122.22, 119.89, 49.37, 41.29, 28.78, 19.80. MS calcd for C_10_H_13_Cl_2_N, 217. ESI-MS: 218.0 (M + H)^+.^

Synthesis of Compound **5**. To a solution of 1-(tert-butoxycarbonyl) piperidine-4-carboxylic acid (17.40 g, 0.076 mol) in dichloromethane (DCM, 100 mL) was added pyridine (18.01 g, 0.23 mol). Then, thionyl chloride (12 mL, 0.11 mol) was added dropwise under an atmosphere of nitrogen and stirred at room temperature for 30 min. Next, the reaction mixture was added **4** (18.20 g, 0.084 mol), triethylamine (Et_3_N, 40 mL, 0.29 mol), and dimethyl aminopyridine (DMAP, 1.12 g, 0.009 mol) in dichloromethane (DCM, 100 mL), followed by stirring at room temperature for another 32 h. The mixture was diluted with 5% aqueous HCl (50 mL) and extracted with dichloromethane (DCM, 3 × 100 mL). The combined organic layer was washed with brine (2 × 100 mL), dried over MgSO_4_, filtered, and concentrated in vacuo. Purification silica gel chromatography (EtOAc/MeOH 1:0 to 5:1) afforded compound **5** as a white solid (9.76 g, 30.04% yield). ^1^H NMR (400 MHz, chloroform-*d*) δ 7.32 (d, *J* = 8.0 Hz, 1H), 7.19 (d, *J* = 2.2 Hz, 1H), 6.99 (dd, *J* = 8.0, 2.2 Hz, 1H), 4.05 (s, 4H), 3.82–3.72 (m, 2H), 3.54 (t, *J* = 6.6 Hz, 2H), 2.87 (t, *J* = 12.3 Hz, 1H), 2.44 (s, 3H), 2.08–1.96 (m, 2H), 1.79–1.64 (m, 2H), 1.60–1.51 (m, 2H), 1.44 (s, 9H). ^13^C NMR (100 MHz, chloroform-*d*) δ 174.73, 154.73, 141.04, 136.69, 135.40, 132.12, 128.49, 126.38, 79.60, 47.67, 42.39, 39.63, 30.90, 28.50, 19.89. MS calcd for C_21_H_30_Cl_2_N_2_O_3_, 428. ESI-MS: 451.2 (M + Na)^+^.

Synthesis of Compound **7**. A mixture of compound **6** (2.23 g, 8.77 mmol), **5** (4.52 g, 10.52 mmol), KI (1.70 g, 10.52 mmol), and K_2_CO_3_ (4.85 g, 35.08 mmol) in DMF/acetonitrile (100 mL, 1:1, *v*/*v*) was stirred at reflux for 18 h. After cooling to room temperature, the mixture was filtered, and the filtrate was concentrated in vacuo. The residue was diluted with water (100 mL) and extracted with EtOAc (3 × 100 mL). The combined organic layer was washed with brine (2 × 100 mL), dried with MgSO_4_, filtered, and concentrated in vacuo. The residue was purified by column chromatography on silica gel (EtOAc/MeOH 1:0 to 7:3) to afford **7** (2.84 g, 53.08%) as a pale-yellow oil. ^1^H NMR (400 MHz, acetone-*d6*) δ 7.91–7.87 (m, 2H), 7.88 (d, *J* = 8.2 Hz, 2H), 7.46 (d, *J* = 15.2 Hz, 1H), 7.45 (d, *J* = 25.3 Hz, 1H), 7.31 (d, *J* = 10.3 Hz, 1H), 7.30 (d, *J* = 8.3 Hz, 2H), 3.96 (d, *J* = 13.3 Hz, 2H), 3.80 (t, *J* = 6.5 Hz, 2H), 3.67 (d, *J* = 12.1 Hz, 2H), 3.23 (s, 2H), 2.99 (d, *J* = 11.7 Hz, 3H), 2.67 (d, *J* = 7.0 Hz, 2H), 2.57–2.42 (m, 2H), 2.39 (s, 3H), 2.11–2.00 (m, 2H), 1.98–1.85 (m, 2H), 1.76–1.65 (m, 3H), 1.63 (s, 2H), 1.63–1.50 (m, 2H), 1.41 (s, 9H). ^13^C NMR (100 MHz, acetone-*d6*) δ 175.23, 168.04, 154.07, 143.44, 141.04, 136.31, 134.70, 132.44, 132.20, 129.01, 128.60, 127.71, 127.26, 78.57, 54.17, 52.45, 46.19, 41.64, 39.32, 35.65, 27.70, 22.47, 18.87. MS calcd for C_34_H_47_ClN_4_O_4_, 610. ESI-MS: 611.3 (M + H)^+^.

Synthesis of TAKW. To an ice-cooled stirred mixture of **7** (2.84 g, 4.65 mmol) in DCM (30 mL) was added 4 N HCl/EtOAc (15 mL), and the mixture was stirred at room temperature for 6 h. The mixture was concentrated in vacuo, diluted with water (20 mL), and 25% ammonia was added to adjust the pH to 9–10. The resulting mixture was extracted with DCM (3 × 50 mL). The combined organic layer was washed with brine (2 × 100 mL), dried with MgSO_4_, filtered, and concentrated in vacuo. Then, glycolic anhydride (0.52 g, 4.41 mmol) in THF (50 mL) was added, and the mixture was stirred at room temperature for 8 h. The residue was concentrated in vacuo and was purified by column chromatography (EtOAc/MeOH 1:0 to 5:1) to afford TAKW (2.84 g, 53.08%) as a yellow oil bubble solid. ^1^H NMR (400 MHz, DMSO-*d6*) δ 9.20 (s, 1H), 7.93 (s, 2H), 7.83 (d, *J* = 8.2 Hz, 2H), 7.56 (d, *J* = 2.2 Hz, 1H), 7.48 (d, *J* = 8.0 Hz, 1H), 7.31 (s, 1H), 7.27 (d, *J* = 8.1 Hz, 2H), 4.30–4.19 (m, 4H), 4.18 (d, *J* = 13.9 Hz, 2H), 4.08 (d, *J* = 2.1 Hz, 4H), 3.44 (d, *J* = 11.8 Hz, 2H), 3.22 (s, 1H), 3.06–2.96 (m, 2H), 2.84 (q, *J* = 11.9 Hz, 2H), 2.60 (d, *J* = 6.6 Hz, 2H), 2.41 (d, *J* = 9.5 Hz, 1H), 2.38 (s, 3H), 2.35 (s, 1H), 1.85–1.71 (m, 5H), 1.60 (s, 2H), 1.38 (q, *J* = 12.0, 11.4 Hz, 2H). ^13^C NMR (100 MHz, DMSO-*d6*) δ 173.69, 171.33, 167.73, 166.84, 142.86, 140.60, 135.73, 133.97, 132.21, 128.91, 128.42, 127.54, 127.24, 69.14, 67.72, 53.52, 51.82, 45.89, 43.26, 41.07, 38.55, 34.69, 28.78, 28.54, 27.93, 22.01, 19.27. MS calcd for C_33_H_43_ClN_4_O_6_, 626. ESI-MS: 627.3 (M + H)^+^.

### 2.2. Peptide Synthesis

Peptides were synthesized on a Liberty Blue peptide synthesizer (CEM, Matthews, NC, USA) via Fmoc solid-phase peptide synthesis (SPPS) protocols on a 0.1 mmol scale using Rink Amide resin of 0.53 mmol/g substitution (RAM; RAPP Polymere, Xi’an, China). To achieve the conjugation of small molecule to peptides, we added an Fmoc-Lys(Dde)-OH to the C-terminus of each peptide. The C-terminus of the peptide taken from the synthesizer was aminated, providing the free amino group at the N-terminus. Acetylation was achieved using an acetic acid anhydride/DIEA mixture [1:1 (*v*/*v*)] (2 × 15 min). Then, the resin was treated with 2% (*v*/*v*) hydrazine hydrate/DMF (5 × 3 min) to remove the Dde protecting group. For the peptide-small molecule coupling reaction, a DMF solution of three equivalents of small molecules, three equivalents of HBTU, three equivalents of HOBt, and six equivalents of DIEA was added to the resin-bound peptide, and the mixture was stirred for 1.5 h at room temperature. The resin was then washed thoroughly with DMF and DCM in turn. The peptides were fully deprotected and cleaved from the resin by treatment with a cleavage cocktail of TFA/thioanisole/*m*-cresol/water/ethanedithiol (16.5:1:1:1:0.5). Crude peptides were precipitated using cold anhydrous ethyl ether and lyophilized in vacuo. The crude products were purified by reverse-phase high-performance liquid chromatography (RP-HPLC). Each compound was confirmed to have ≥95% purity by analytical HPLC. The molecular weight of the peptides was confirmed by MALDI-TOF-MS (Autoflex III, Bruker Daltonics, Billerica, MA, USA).

### 2.3. HIV-1 Infection Assay

To measure the inhibitory activity of the peptides on the infection of the laboratory-adapted HIV-1 Bal strain, primary HIV-1 isolates, and T20-resistant strains, 1 × 10^5^ CEMx174 5.25M7 cells in RPMI 1640 medium containing 10% fetal bovine serum (FBS) were infected with 100 TCID50 of a virus in the presence of different concentrations of inhibitors. On the fourth day post-infection, fifty-microliter amounts of the culture supernatants were collected and mixed with equal volumes of 5% Triton X-100. The p24 antigen was detected using an enzyme-linked immunosorbent assay (ELISA). Percent inhibition by the peptides and 50% effective concentration (EC_50_) values were calculated using Calcusyn software (Biosoft, Ferguson, MO, USA).

### 2.4. Cytotoxicity Assay

The potential cytotoxicity of AP3P4E on the CEMx174 5.25M7 target cell used for the viral inhibition assays in this study was measured by a standard Cell Counting Kit-8 (CCK8) assay. Serial dilutions of AP3P4E were mixed with CEMx174 5.25 M7 cells. Then, the culture medium was replaced with fresh RPMI 1640 medium with 10% FBS 10 h later. The cytotoxicity of AP3P4E was tested after an additional 48 h by using the Cell Counting Kit-8 (Dojindo, Kumamoto, Japan).

### 2.5. Circular Dichroism (CD) Spectroscopy

N36 and AP3 or AP3P4E were dissolved in ddH_2_O and PBS (1×, pH 7.4), respectively, at a final concentration of 10 μM. Equimolar mixtures were incubated at 37 °C for 30 min. CD spectra of each sample were recorded on a Chirascan Plus qCD (Applied Photophysics, Leatherhead, UK) using a 1 nm bandwidth with a 1 nm step resolution from 195 to 260 nm at room temperature. The spectra were corrected by subtracting a blank corresponding to the solvent composition of each sample. The CD data are shown as the mean residue ellipticity, and the mean residue ellipticity at 222 nm ([θ]_222nm_) divided by the expected value of 100% α-helix formation (−33,000° cm^2^/dmol) was calculated to obtain the α-helical content. The thermal denaturation experiment was performed by applying a thermal gradient of 2 °C/min and monitoring the change in ellipticity from 20 °C to 90 °C at 222 nm.

### 2.6. Native Polyacrylamide Gel Electrophoresis (N-PAGE)

Each peptide was dissolved in PBS (1×, pH 7.4) to a final concentration of 100 μM. Equimolar mixtures of N36 and the test peptides were incubated at 37 °C for 30 min. After mixing the above peptides with 5× methyl green loading buffer (1:4, *v*/*v*), the samples were loaded (15 μL in each well) onto a Basic Protein Native PAGE Gel (8%). Gel electrophoresis was carried out at a constant voltage of 120 V for 4 h at room temperature. The obtained gel was stained with Coomassie Blue R250 and imaged with a ChampGel 6000 imaging system (Sage Creation Ltd., Beijing, China).

### 2.7. Calcium Mobilization Assay

HEK293 cells (20,000 cells per well) stably expressing Gα15 and CCR5 were seeded into wells of a polyD-lysine-coated black-wall, clear bottom 384-well plate and cultured for 20 h at 37 °C and 5% CO_2_. Then, 20 μL of 2× Fluo-4 Direct™ Calcium Assay Buffer was added from the kit directly to the wells, followed by incubation at 5% CO_2_ and 37 °C for 50 min and 10 min at room temperature. The antagonist (10 μL at varying concentrations) was added to wells and preincubated for 1 min prior to the addition of the RANTES-(CCL5) (10 μL at a final concentration of 34.62 nM). The intracellular calcium change was recorded by the Fluorometric Imaging Plate Reader (FLIPR) at 494 nm (excitation)/516 nm (emission). Data were analyzed by the GraphPad Prism 9.0 Program.

### 2.8. Solubility

The peptide (1 mg) was weighed in LoBind Eppendorf tubes, and 10 μL of dd-H_2_O or PBS (pH 7.4) was added to dissolve the samples. After initial mixing using brief vortexing and sonication (5–10 min), saturated solutions of compounds in dd-H_2_O or PBS were shaken at room temperature for 24 h. Suspensions were filtered through 0.45 μm PVDF membrane filters, and the dissolved drug concentration was analyzed using an RP-HPLC assay. Aqueous concentration was determined by comparison of the peak area of the saturated solution with a standard curve plotted for the peak area versus the known concentrations. Each sample was performed in triplicate.

### 2.9. Metabolic Stability

Animals were treated in accordance with the Animal Welfare Act and the Guide for the Care and Use of Laboratory Animals (NIH Publication 86-23, Revised 1985). The metabolic stability of peptides was evaluated with proteinase K, rat plasma, or tissue homogenate. Peptides were diluted in proteinase K solution (100 ng/mL), plasma, and tissue homogenate, respectively, to a final concentration of 50 μM and incubated at 37 °C. At different timepoints (0, 5, 15, 30, 60, 120, and 240 min), the solution (50 μL) was added to 50 μL of acetonitrile containing 0.1% TFA to stop the reaction. The samples were centrifuged for 10 min, and the supernatant was analyzed by HPLC. The amount of peptide residue in the mixture was estimated by integrating the area under the corresponding elution peak monitored at 210 nm.

## 3. Results and Discussion

### 3.1. Design and Chemistry

**Figure 2:**
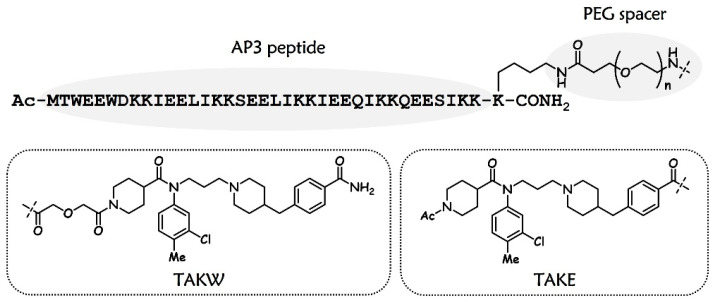
Design strategy of dual-target ligands. The letter “n” represents the number of PEG units in the linker.

As the major coreceptor for HIV-1 infection, CCR5 is naturally an attractive target for HIV-1 entry inhibitor research. A decade after uncovering the critical role of CCR5 in mediating HIV-1 entry, the first-in-class CCR5 antagonist known as maraviroc was approved in 2007 to provide an addition to the anti-HIV-1 treatment arsenal [22,23]. More encouragingly, many other promising CCR5 antagonists have been discovered and have entered clinical development. For example, the piperidine-4-carboxamide chemokine CCR5 antagonist TAK-220, which was discovered by Takeda Pharmaceutical Co., Ltd. (Osaka, Japan), was ultimately selected as a clinical candidate and has entered Phase I clinical trials [24]. Based on the known structural tolerance at the acetyl group of the left-side piperidine ring of TAK-220 [25], dual-target inhibitors were initially engineered by the introduction of the free-acid form of TAK-220, designated TAKW (westerly connection), to the side chain of a lysine residue that was appended to the C-terminus of an artificial peptide, AP3, through different lengths of polyethylene glycol (PEG) spacers. With the optimal linker length in place, we next focused on the optimization of the attachment point of the CCR5 antagonist through the incorporation of TAKE (easterly connection). The selection of the linkage site for TAKE at the carbamoyl group of the right-side phenyl ring of TAK-220 was based on previously concluded structure–activity relationships (Figure 2) [26].

**Scheme 1:**
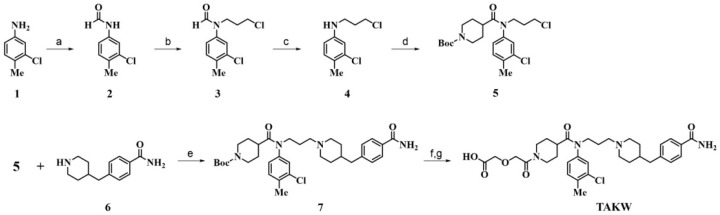
Synthesis of the CCR5 coreceptor antagonist component TAKW. Reagents and conditions: (a) acetic anhydride, formic acid, 60 °C; (b) 1-bromo-3-chloropropane, Cs_2_CO_3_, acetone, reflux; (c) HCl, *i*-PrOH; (d) *N*-Boc-piperdine-4-carboxylic acid, SOCl_2_, TEA, DCM, rt; (e) KI, K_2_CO_3_, DMF/MeCN (1:1, *v*/*v*), 80 °C; (f) TFA, DCM; and (g) glycolic anhydride, THF, rt.

The synthesis of TAKW free acid is described in Scheme 1. Commercially available aniline **1** was protected with acetic formic anhydride to provide formamide **2**, which underwent *N*-alkylation by use of 1-bromo-3-chloropropane, followed by the removal of the formyl group to provide *N*-(3-chloropropyl)aniline **4**. Subsequent reaction with *N*-Boc-piperidine-4-carboxylic acid gave compound **5**, which was subjected to a further coupling with the *N*-substituted piperidine **6** in the presence of potassium iodide and potassium carbonate to afford **7**. After acidic deprotection, the unprotected piperidine was converted to building block TAKW by reaction with glycolic anhydride. Building block TAKE was prepared according to the synthetic route shown in our previous study [20]. Preparation of bifunctional entry inhibitors began with the introduction of a lysine with a Dde side chain-protecting group at the C-terminus of the AP3 peptide achieved by the use of Fmoc solid-phase peptide synthesis (SPPS). After site-specific deprotection at the lysine side chain ε-amino group, the sequential incorporation of the PEG linker and modified version of TAK-220 into peptides was performed by an on-resin reaction using HBTU and 1-hydroxybenzotriazole (HOBt) as coupling reagents.

### 3.2. Potent Antiviral Activity of Artificially Designed Peptides against HIV-1 Strains

We first tested the anti-HIV-1 activities of these dual-target ligands in comparison with AP3, TAK-220, and T20. As shown in Table 1, the initial hit compound AP3 inhibited the authentic HIV-1 Bal strain with a 50% effective concentration (EC_50_) value of 0.75 nM. The CCR5 antagonist TAK-220 had an EC_50_ value of 23.8 nM. The hybrid molecule AP3W, consisting of TAKW joined to the C-terminus of AP3 without a linker, inhibited virus replication with an EC_50_ value of 6.33 nM, indicating an 8.4-fold decrease in potency relative to that of the parent AP3 moiety. However, AP3P4W, in which a 4-unit PEG linker is introduced between the peptide sequence and the CCR5 antagonist, could inhibit HIV-1 Bal infection similarly relative to AP3. Moreover, AP3P8W had activity similar to that of AP3P4W. It was found that the potency of inhibitors decreased upon further extension of the spacer length, as shown by the EC_50_ values of AP3P12W, AP3P16W, and AP3P24W. These results showed that linker length exerted considerable influence on the overall activity of the chimera compounds. On the basis of the structures of AP3P4W and AP3P8W, AP3P4E and AP3P8E were constructed by replacing the TAKW fragment with TAKE. Strikingly, the EC_50_ value of AP3P4E for inhibiting virus infection was 0.15 nM, which is 26-fold more potent than that of T20. In addition, this chimera exhibited antiviral potency much stronger than either of the pharmacophores alone or the unlinked 1:1 mixture of AP3 and TAK-220. These findings suggest that the covalent linkage of both compounds into one molecule is essential for enhanced activity. To assess cellular safety, we evaluated AP3P4E in a standard Cell Counting Kit-8 (CCK8) assay. It was found that compound AP3P4E showed no cytotoxicity at a concentration of 2000 nM on the CEMx174 5.25M7 cells that were used for the viral inhibition assay in our study (Figure S2). Besides the laboratory-adapted strain HIV-1 Bal, we also tested artificial AP3P4E against a panel of primary HIV-1 isolates with distinct genotypes and phenotypes. As shown in Table 2, it inhibited HIV-1 91US_4 (subtype B, R5) with an EC_50_ of 3.82 nM compared to 28.2 nM for T20. AP3P4E also blocked infection by HIV-1 J32228M4 (subtype D, R5) with an EC_50_ of 8.86 nM compared to 59.0 nM for AP3 and 161 nM for TAK-220. Encouragingly, testing on clinical HIV-1 strain 89BZ167 (subtype B, X4) exhibited 3.7- and 15.6-fold improvement by AP3P4E over AP3 and T20, respectively. In addition, AP3P4E displayed 6.7-fold more potency than T20 in inhibiting infection by HIV-1 92UG029 (subtype A, X4). In sharp contrast, TAK-220 alone showed no inhibitory activity at a concentration up to 300 nM. The administration of the CCR5 antagonist for the treatment of HIV-1 infection has been limited by the requirement for a diagnostic viral tropism test and its failure to prevent the infection of CXCR4-utilizing strains, the emergence of which correlates with worsening immunodeficiency [27,28,29]. The high antiviral activity of this chimeric inhibitor against both R5- and X4-tropic virus strains overcomes the major drawback of the parent CCR5 coreceptor antagonist. Lipid conjugation is a widely used strategy for improving the antiviral activity of fusion-inhibitory peptides. This is mainly achieved through increasing the effective fusion inhibitor concentration at viral entry sites on the target cell membrane surface and overcoming the diffusion-limited association rate for inhibitors with their gp41 target [30,31,32,33,34]. By modifying peptide fusion inhibitors with different lipids such as fatty acid and cholesterol, we and others identified a large panel of membrane-anchoring lipopeptides with enhanced antiviral activity. Very recently, we dedicated our efforts to developing a new multitarget design concept to create dual-function inhibitors that can hit both the CCR5 chemokine receptor located on the host cell membrane surface and HIV-1 Env gp41. Different to localizing inhibitors to cell membranes via lipid cargoes, we joined a small-molecule CCR5 antagonist to a fusion-inhibitory peptide, termed C34, by a flexible tether. This resulted in a strong efficacy profile caused by the synergistic inhibition of HIV-1 entry by the two drugs, and the resultant CP12TAK potently inhibited HIV-1 infection. Disappointingly, the poor biophysical properties of C34 (e.g., low aqueous solubility [11]) severely complicate its medicinal use, and, unexpectedly, the C34 peptide-based dual-target inhibitor CP12TAK suffered from adverse water solubility. Nonetheless, the data presented herein show that the addition of a CCR5 antagonist to the C-terminus of the artificial peptide AP3 could markedly increase the antiviral activities of inhibitors, validating the general role of the multitarget-directed ligands concept in the design or optimization of new-generation HIV-1 entry inhibitors. Meanwhile, compared to CP12TAK, the much higher aqueous solubility of these multi-functional artificial peptide entry inhibitors make them suitable for further drug development (Table 3).

### 3.3. Antiviral Activities of AP3P4E against T20-Resistant HIV-1 Variants

T20 has a relatively low genetic barrier for the evolution of resistant viruses. The rapid emergence and spread of resistant HIV-1 isolates has caused an increasing number of patients who fail to respond to T20 treatment. One of the primary goals of creating a new generation of HIV-1 fusion inhibitors is to avoid cross-drug resistance with the currently used drug T20. As shown in Table 4, mutations in the NHR region of HIV-1 virus NL4-3 conferred a high level of resistance to T20. In contrast, T20-resistant variants still remained sensitive to this artificially designed peptide inhibitor. For example, the V38A/N42T double mutation resulted in a 941.4-fold resistance for T20, but it did not affect the inhibitory activity of AP3P4E. Similarly, the impact of N42T/N43K and V38E/N42S T20-resistant mutations was also absorbed by the chimeric entry inhibitor. Despite its efficacy against the T20-resistant strains, one may question whether this multitargeted compound can induce drug-resistance. Previously, Chong et al. demonstrated that introducing an M-T hook motif into the N-terminus of a peptide HIV-1 fusion inhibitor (i.e., sifuvirtide), could significantly increase its genetic barrier to drug resistance [35]. Consistently, Xue et al. described a membrane-localizing, cholesterol-tagged HIV-1 fusion inhibitory peptide named LP-98, which failed to select resistant viruses either in vitro or in vivo [6]. These results indicate that the development of a bifunctional chimera through the covalent combination of two drugs with different target sites and mechanisms of action may be an effective way to overcome the rapid emergence of resistant virus mutations since co-evolution of both resistance pathways may affect viral fitness.

### 3.4. Interaction of AP3P4E with an Exogenous gp41 NHR Peptide

**Figure 3:**
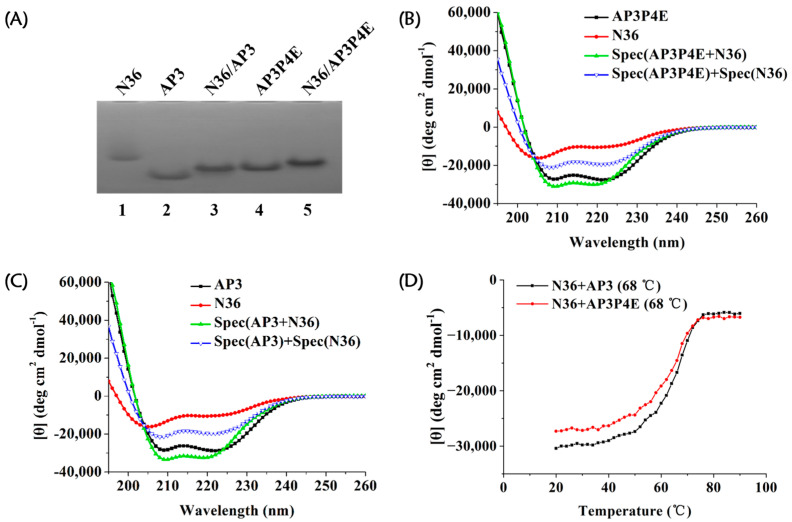
Interaction of the chimeric inhibitor AP3P4E with a gp41 NHR-derived peptide. (**A**) Visualization of binding between AP3P4E and the NHR peptide N36 by native PAGE. The final concentration of each peptide was 100 μM. The α-helical conformation of the complexes formed by (**B**) AP3P4E or (**C**) AP3 and N36 was analyzed by CD spectroscopy. The CD spectrum of peptide mixtures (Spec(C + N), solid symbols) and the sum of the spectra of related isolated peptides (Spec(C) + Spec(N), open symbols) are shown for comparison. The final concentration of each peptide in PBS (pH 7.4) was 10 μM. (**D**) Thermostability of the AP3P4E/N36 and AP3/N36 complexes in the presence of 2 M Gdn·HCl. Each peptide was used at a final concentration of 10 μM.

First, we used a native PAGE (N-PAGE)-based method to evaluate the capacity of the bifunctional entry inhibitor AP3P4E to bind a synthetic peptide derived from the NHR segment of HIV-1 gp41, namely, N36. As shown in Figure 3A, the gp41 NHR target surrogate N36 peptide, artificially designed peptide AP3, and AP3P4E displayed specific bands at different positions in the gel depending on their net positive charges and molecular size. Both AP3 and AP3P4E bound to N36 in equimolar mixtures, as inferred from the appearance of new bands concomitant with the disappearance of AP3 and AP3P4E bands. Subsequently, the CD spectra and thermostability of the AP3P4E/N36 complex were analyzed. Isolated AP3 and AP3P4E exhibited a similar α-helical structure with a helical content of 86% and 83%, respectively. The N36 peptide was in the α-helical conformation with 32% helicity. Obviously, the α-helicity of an equimolar mixture of AP3P4E and N36 (91%) was much higher than that of the sum of the CD signals of these two single peptides (60%) (Figure 3B). The difference between the two spectra indicated that the interaction between AP3P4E and N36 resulted in the enhanced helical structures of the component peptides [36]. This is consistent with the results from the CD mixing experiment of the AP3/N36 pair (Figure 3C). In addition, data from the CD analysis indicated that both the AP3P4E/N36 and AP3/N36 complexes had undergone thermal unfolding transition (*Tm*) values of 68 °C (Figure 3D). These results suggest that the new chimeric molecule AP3P4E can function like its parent compound AP3 by binding to the gp41 N-trimer and blocking the formation of fusogenic gp41 6-HB.

We previously reported that a 1:1 noncovalent mixture of C34 and TAK-220, which mimicked the molar ratio between the inhibitors in the CP12TAK chimera, showed an EC_50_ value of 0.58 nM, similar to that of T20 (EC_50_ = 1.58 nM). In the present study, we discovered that the mixture of AP3/TAK-220 at equimolar concentration also showed comparable activity with T20 (EC_50_: 8.02 nM vs. 4.02 nM). The AP3P4E could effectively inhibit HIV-1 strain Bal infection with an EC_50_ value of 0.15 nM, about 53-fold more potent than the 1:1 (mol/mol) noncovalent mixture of AP3/TAK-220, whereas CP12TAK showed about a 19-fold enhanced potency compared with that of the equimolar mixture of C34 with TAK-220. The circular-dichroism (CD) spectroscopic data showed that the AP3 peptide formed a typical α-helical conformation, while C34 adapted a largely random coil structure in solution [20]. Therefore, one possible explanation of the less potency enhancement of the chimeric inhibitor CP12TAK than AP3P4E against the Bal strain is that the transient intramolecular interaction between peptide fusion inhibitor C34 and small-molecule CCR5 inhibitor TAK-220 may shield their target binding sites. In contrast, the rigid rod-like α-helix of AP3 could facilitate each pharmacophore in AP3P4E to bind to its respective target, thus causing a stronger synergistic anti-HIV-1 effect.

### 3.5. Antagonistic Activity of AP3P4E on RANTES-Induced Ca^2+^ Mobilization in CCR5-Expressing Cells

**Figure 4:**
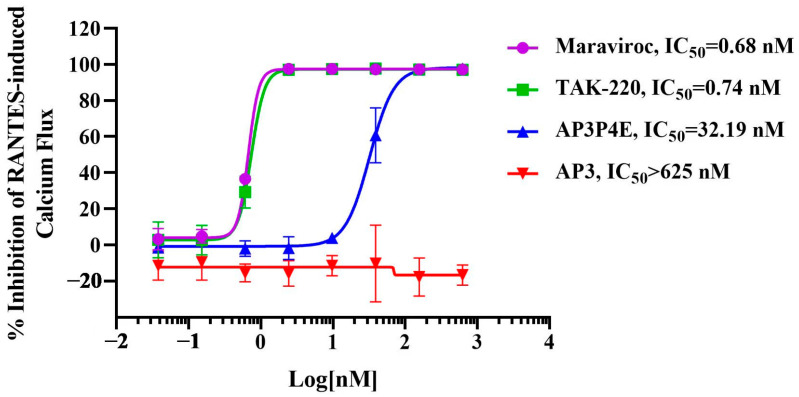
Assessment of the inhibitory effect of HIV-1 entry inhibitors on RANTES-induced Ca^2+^ mobilization in CCR5-expressing HEK293 cells. Cells loaded with Fluo-4 were treated with serial dilutions of HIV-1 inhibitors followed by stimulation with RANTES at 34.6 nM. Experiments were performed in duplicate, and the data expressed as means ± the standard deviations (error bar). Percent inhibition of the compounds and IC_50_s were calculated.

We then conducted a calcium mobilization assay to test the CCR5 coreceptor-inhibitory function of the newly designed chimera AP3P4E in HEK293 cells with maraviroc as a positive control. As shown in Figure 4, both TAK-220 and maraviroc showed potent inhibitory activity against CCR5 with half-maximal inhibitory concentration (IC_50_) values of 0.74 nM and 0.68 nM, respectively. As expected, AP3 exhibited no significant inhibitory activity at the concentration up to 625 nM. Compound AP3P4E containing the TAK-220 pharmacophore retained CCR5 antagonistic activity, as judged by the IC_50_ value of 32.19 nM. Combined with the N-PAGE and CD data, these experiments provide evidence indicating that the high potency of this bifunctional entry inhibitor results from cellular CCR5 binding, along with its ability to target HIV-1 gp41.

### 3.6. High Resistance of AP3P4E to Proteolytic Degradation

**Figure 5:**
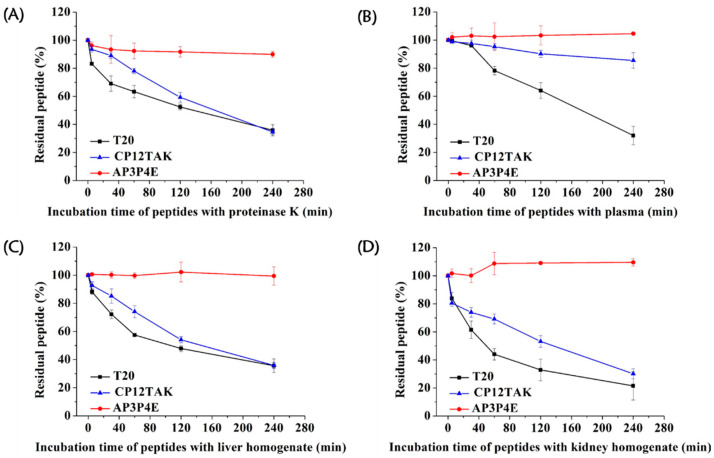
Metabolic stability of AP3P4E, CP12TAK, and T20 (50 μM) in (**A**) proteinase K solution, (**B**) rat plasma, (**C**) liver homogenate, and (**D**) kidney homogenate.

Although the T20 peptide has potent anti-HIV-1 activity, it suffers from susceptibility to enzymatic degradation. This is nicely illustrated by the fact that T20 treatment requires subcutaneous injection of a dosage of 90 mg of the peptide twice daily in order to maintain an effective therapeutic concentration [37]. Therefore, it would be highly interesting to know whether the α-helical peptide AP3P4E is more resistant to proteolytic degradation than T20. To make this determination, degradation experiments were first performed over a time frame of 240 min by incubating AP3P4E or T20 with proteinase K (a broad-spectrum serine proteinase). As shown in Figure 5A, AP3P4E maintained 89.9% of the original amount in the proteinase K after a 4-h incubation. In sharp contrast, under the same conditions, T20 retained only 35.8% of the original amount. Similarly, AP3P4E remained essentially intact after a 4-h incubation in rat plasma while T20 showed 68.0% degradation (Figure 5B). Next, the proteolytic stability of peptides AP3P4E and T20 in the liver and kidney homogenates was monitored by HPLC analysis. Nearly all of the T20 peptide was digested after 4-h in the presence of homogenates, whereas AP3P4E maintained most of its original amount in the liver and kidney homogenates (Figure 5C,D). The above-mentioned results showed that the bifunctional HIV-1 entry inhibitor displayed greater proteolytic stability than T20 and that such improvement in biophysical properties may contribute to its potential as a drug candidate for clinical development. As shown in Figure 5A, CP12TAK was more susceptible to proteinase K degradation as about 11% of the peptide was degraded at 0.5 h. Additionally, CP12TAK showed 24.9% degradation after a 4 h incubation in plasma, while AP3P4E remained essentially intact (Figure 5B). Furthermore, after a 4-h treatment in the liver and kidney homogenates, CP12TAK retained only 35.8% and 30.1% of the original amount, respectively (Figure 5C,D). These results indicate that the newly engineered AP3P4E is much more resistant to proteolytic degradation than CP12TAK. Although previously discovered chimera CP12TAK exhibits highly potent anti-HIV-1 activity, this compound is not druggable because of its high proteolytic susceptibility and low aqueous solubility (Table 3). In this study, we performed optimization and structure–activity relationship studies on the bifunctional entry inhibitor scaffold that resulted in AP3P4E with improved drug-like properties to meet the clinical needs.

## 4. Conclusions

In conclusion, to overcome the shortcomings of T20, we designed and engineered an artificial peptide-based bifunctional HIV-1 entry inhibitor, AP3P4E, by adding a CCR5-binding small molecule to an artificially designed gp41-binding peptide. Results showed that the incorporation of two inhibitors into one molecule can result in significantly improved antiviral capabilities compared with that of either individual component alone. In addition, this chimeric inhibitor presented a dual mechanism of action involving HIV-1 envelope gp41 NHR-binding and cellular coreceptor CCR5 antagonism. Compared with T20, the hybrid compound is much more potent in blocking infection by both the laboratory-adapted HIV-1 strain Bal and a broad spectrum of primary isolates of HIV-1. Promisingly, AP3P4E possessed high potency against T20-resistant HIV-1 strains and exhibited significantly enhanced aqueous solubility and reduced sensitivity to proteolytic degradation when compared to T20. These attributes make AP3P4E an ideal drug candidate for further development. Moreover, this study provides convincing data for our proposed concept that the MTDLs approach can serve as a viable strategy for designing novel HIV-1 entry inhibitors.

## Figures and Tables

**Table 1:** Inhibition of dual-target entry inhibitors on laboratory-adapted HIV-1 R5 virus infection ^a^.

Compound	Sequence ^b^	EC_50_ (nM)
AP3	MTWEEWDKKIEELIKKSEELIKKIEEQIKKQEESIKK	0.75 ± 0.22
AP3W	MTWEEWDKKIEELIKKSEELIKKIEEQIKKQEESIKK(TAKW)	6.33 ± 1.88
AP3P4W	MTWEEWDKKIEELIKKSEELIKKIEEQIKKQEESIKK(PEG4-TAKW)	0.98 ± 0.23
AP3P8W	MTWEEWDKKIEELIKKSEELIKKIEEQIKKQEESIKK(PEG8-TAKW)	0.53 ± 0.17
AP3P12W	MTWEEWDKKIEELIKKSEELIKKIEEQIKKQEESIKK(PEG12-TAKW)	2.41 ± 0.39
AP3P16W	MTWEEWDKKIEELIKKSEELIKKIEEQIKKQEESIKK(PEG16-TAKW)	4.03 ± 0.76
AP3P24W	MTWEEWDKKIEELIKKSEELIKKIEEQIKKQEESIKK(PEG24-TAKW)	8.98 ± 0.91
AP3P4E	MTWEEWDKKIEELIKKSEELIKKIEEQIKKQEESIKK(PEG4-TAKE)	0.15 ± 0.08
AP3P8E	MTWEEWDKKIEELIKKSEELIKKIEEQIKKQEESIKK(PEG8-TAKE)	0.29 ± 0.16
TAK-220	-	23.8 ± 13.3
AP3/TAK-220 ^c^	-	8.02 ± 3.00
T20	YTSLIHSLIEESQNQQEKNEQELLELDKWASLWNWF	4.02 ± 0.69

^a^ The assay was performed in triplicate, and the data are expressed as the mean ± standard deviation. CEMx174 5.25 M7 cells expressing both CCR5 and CXCR4 on the surface were infected with the HIV-1 BaL strain (subtype B, R5). ^b^ For these peptides, the N-termini were acetylated, and the C-termini were amidated. ^c^ Molar ratio of AP3/TAK-220 in combination was 1:1.

**Table 2:** Inhibitory effect of AP3P4E against infection by the primary HIV-1 isolates ^a^.

HIV-1 Isolate	Subtype	Tropism	EC_50_ (nM) ^b^			
			AP3	TAK-220	AP3P4E	T20
91US_4	B	R5	10.7 ± 1.2	12.1 ± 2.7	3.8 ± 0.3	28.2 ± 2.4
J32228M4	D	R5	59.0 ± 6.1	161.0 ± 6.6	8.9 ± 1.4	59.8 ± 7.1
89BZ167	B	X4	11.6 ± 0.5	>300	3.09 ± 0.03	48.3 ± 2.9
92UG029	A	X4	3.1 ± 0.1	>300	1.53 ± 0.03	10.3 ± 2.3

^a^ All assays were performed in triplicate, and data are expressed as the mean ± standard deviation. ^b^ CEMx 174 5.25 M7 cells expressing both CCR5 and CXCR4 on the surface were used in this study.

**Table 3:** Solubility of bifunctional inhibitors and the T20 peptide ^a^.

Compound	Solubility (mg/mL) in	
	PBS (pH 7.4)	H_2_O
AP3P4E	>60	>60
CP12TAK	1.28 ± 0.04	1.22 ± 0.04
T20	1.02 ± 0.03	0.33 ± 0.01

^a^ The assay was performed in triplicate, and the data are expressed as the mean ± standard deviation.

**Table 4:** Inhibitory activity of AP3P4E against infection by T20-resistant HIV-1 strains ^a^.

HIV-1 Strains	EC_50_ (nM) ^b^	
	T20	AP3P4E
D36G ^c^	1.11 ± 0.23	0.97 ± 0.40
N42T/N43K ^d^	1264.0 ± 45.1 (1138.7)	0.17 ± 0.05 (0.2)
V38E/N42S ^d^	>2000.0 (>1801.8)	2.02 ± 0.39 (2.1)
V38A/N42T ^d^	1045.0 ± 48.6 (941.4)	1.13 ± 0.13 (1.2)

^a^ The assay was performed in triplicate, and the data are expressed as the mean ± standard deviation. Values in parentheses indicate relative changes (n-fold) in the EC_50_ compared with EC_50_ in the presence of the D36G substitution. ^b^ CEMx174 5.25 M7 cells expressing both CCR5 and CXCR4 on the surface were used in this study. ^c^ T20-sensitive strain. ^d^ T20-resistant strain.

## Data Availability

Data can be available from the corresponding authors upon request.

## Supplementary Materials

The following supporting information can be downloaded at: https://www.mdpi.com/article/10.3390/v15051038/s1, Figure S1: Schematic representation of the strategy used for preparation of bifunctional molecules; Figure S2: The potential cytotoxicity of peptide AP3P4E; Table S1: HPLC method used for the purification of peptide compounds; Table S2: HPLC method used for the analysis of peptide compounds; MALDI-TOF-MS and analytical HPLC of designed peptides.

## Abbreviations

CHR: C-terminal heptad repeat; NHR: N-terminal heptad repeat; 6-HB: six-helix bundle; gp120: glycoprotein 120; gp41: glycoprotein 41; FDA: Food and Drug Administration.
