# Supplementary material for: An Artificial Peptide-Based Bifunctional HIV-1 Entry Inhibitor That Interferes with Viral Glycoprotein-41 Six-Helix Bundle Formation and Antagonizes CCR5 on the Host Cell Membrane
Source: Viruses. 2023 Apr 23;15(5):1038. doi: 10.3390/v15051038 (PMC10223265; doi:10.3390/v15051038)

Supporting Information

An Artificial Peptide-based Bifunctional HIV-1 Entry Inhibitor That Interferes with Viral Glycoprotein-41 Six-Helix Bundle Formation and Antagonizes CCR5 on the Host Cell Membrane

Chao Wang,[,†,¶] Qing Li,[†,¶] Lujia Sun,[‡,¶] Xinling Wang,[‡] Huan Wang,[†] Wenpeng Zhang,[†] Jiahui Li,[†,#] Yang Liu,[*,#] Lu Lu,[*,‡] and Shibo Jiang[*,‡]*

[†] State Key Laboratory of Toxicology and Medical Countermeasures, Beijing Institute of Pharmacology and Toxicology, 27 Tai-Ping Road, Beijing 100850, China;
[‡] Key Laboratory of Medical Molecular Virology (MOE/NHC/CAMS), School of Basic Medical Sciences & Shanghai Public Health Clinical Center, Shanghai Institute of Infectious Diseases and Biosecurity, Fudan University, 131 Dong An Road, Shanghai 200032, China;
[#] Key Laboratory of Structure-based Drug Design & Discovery of Ministry of Education, School of Pharmaceutial Engineering, Shenyang Pharmaceutical University, Shenyang 110016, China.

[¶] These authors contributed equally to this work.

Corresponding Authors:

S.J.: Key Laboratory of Medical Molecular Virology of (MOE/NHC/CAMS), School of Basic Medical Sciences & Shanghai Public Health Clinical Center, Fudan University, 131 Dong An Road, Shanghai 200032, China; Tel.: 86-21-5423-7673; Fax: 86-21-5423-7465; E-mail: shibojiang@fudan.edu.cn.

C.W.: State Key Laboratory of Toxicology and Medical Countermeasures, Beijing Institute of Pharmacology & Toxicology, 27 Tai-Ping Road, Beijing 100850, China; Tel.: 86-10-6693-0640; Fax: 86-10-6821-1656, E-mail: chaow301@sina.com.

L.L.: Key Laboratory of Medical Molecular Virology of (MOE/NHC/CAMS), School of Basic Medical Sciences & Shanghai Public Health Clinical Center, Fudan University, 131 Dong An Road, Shanghai 200032, China; Tel.: 86-21-5423-7671; Fax: 86-21-5423-7465; E-mail: lul@fudan.edu.cn

Y.L.: Key Laboratory of Structure-based Drug Design & Discovery of the Ministry of Education, Shenyang Pharmaceutical University, Shenyang 110016, China; Tel.: 86-24-4352-0227; E-mail: y.liu@syphu.edu.cn

Table of Contents

Supplemental Tables and Figures

Table S1 HPLC method used for the purification of peptide compounds.................................... S2
Table S2 HPLC method used for the analysis of peptide compounds...................................... S2
Figure S1 Schematic representation of the strategy used for preparation of bifunctional molecules...... S3
Figure S2 The potential cytotoxicity of peptide AP3P4E... S3

MALDI-TOF-MS and Analytical HPLC of designed peptides... S4

Table S1. HPLC method used for the purification of peptide compounds[a]

Time (min)	Solvent A (%)	Solvent B (%)
5	70	30
13	53	47
30	17	83
35	17	83
55	70	30
60	70	30

[a] The crude peptide products were purified by preparative reverse phase HPLC with a Waters preparative HPLC system (PrepLC 4000) on a Waters X-bridge C8 column (19.5mm × 250mm, 10μm) at constant flow rate of 16 mL/min. Solvent A: 0.1% trifluoroacetic acid in H_2O; Solvent B: 0.1% trifluoroacetic acid in 70% CH_3CN/H_2O.

Table S2. HPLC method used for the analysis of peptide compounds[a]

Methods	Time (min)	Solvent A (%)	Solvent B (%)
Method A	5	50	50
	10	0	100
	15	0	100
	20	0	100
	23	0	100
	25	90	10
Method B	5	50	50
	10	30	70
	15	10	90
	20	0	100
	23	0	100
	25	90	10

[a] The peptide compounds were analyzed by analytical RP-HPLC was performed on a RP-C8 column (Zorbax Eclipse XDB-C8, 4.6 × 150 mm, 5 μm) using two different solvent systems (Methods A and B), and a flow rate of 1 mL/min with detection at 210 nm. Solvent A: 0.1% trifluoroacetic acid in H_2O; Solvent B: 0.1% trifluoroacetic acid in 70% CH_3CN/H_2O.

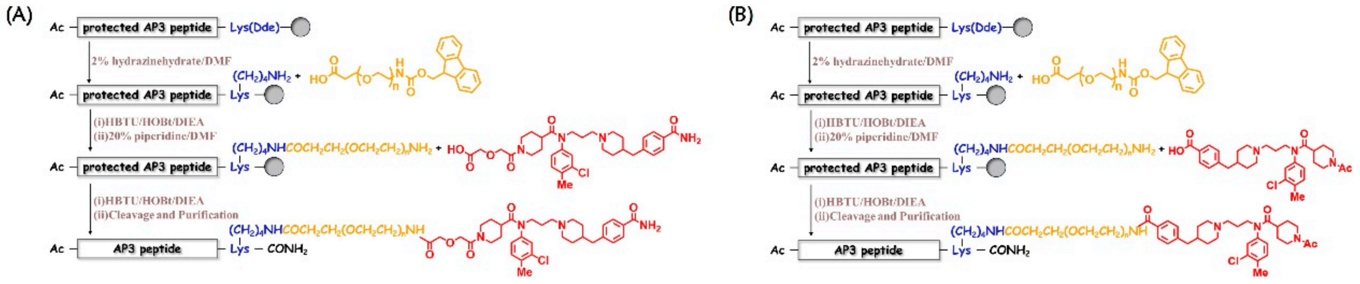

Figure S1. Schematic representation of the strategy used for preparation of bifunctional molecules

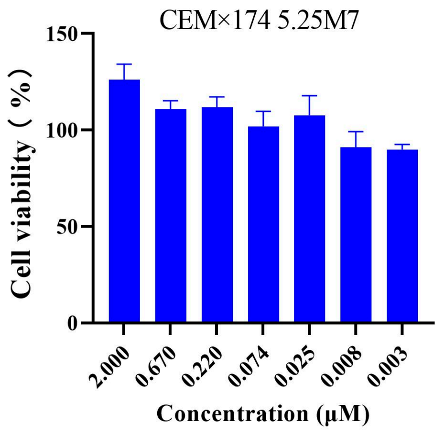

Figure S2. The potential cytotoxicity of AP3P4E on the CEMx174 5.25M7 cells.

MALDI-TOF-MS and analytical HPLC of designed peptides

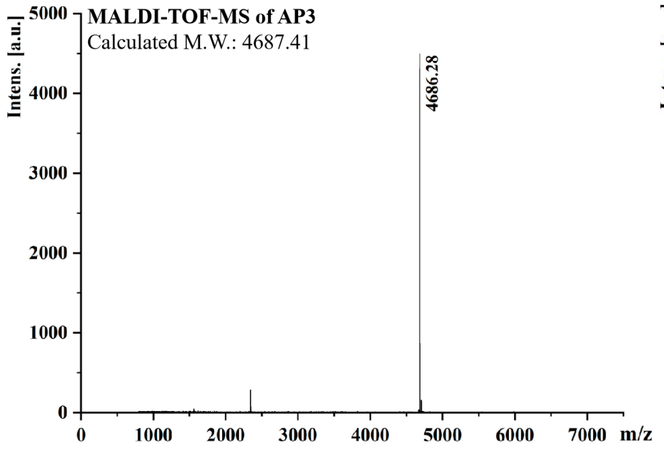

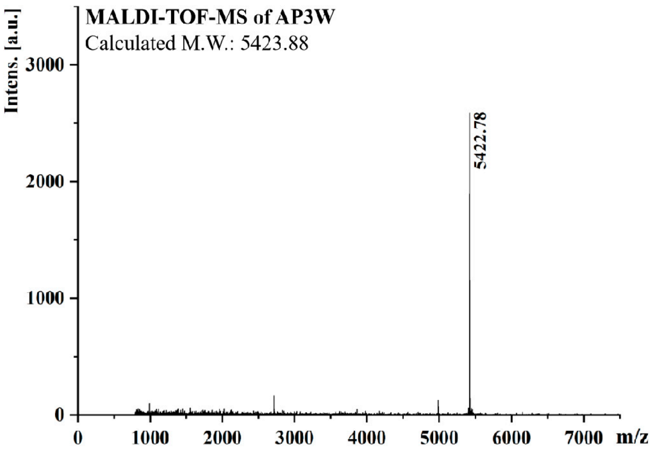

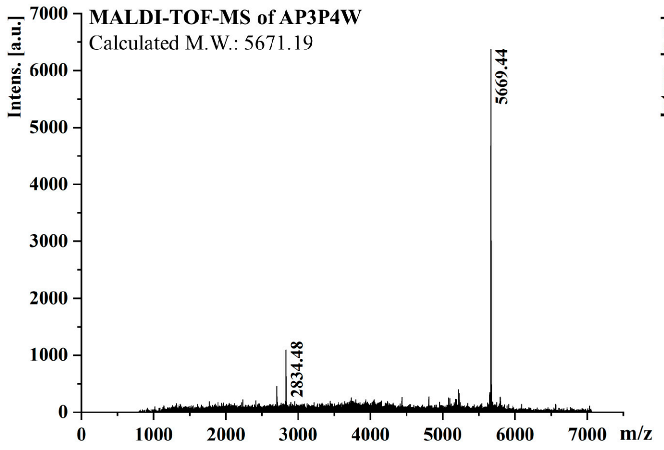

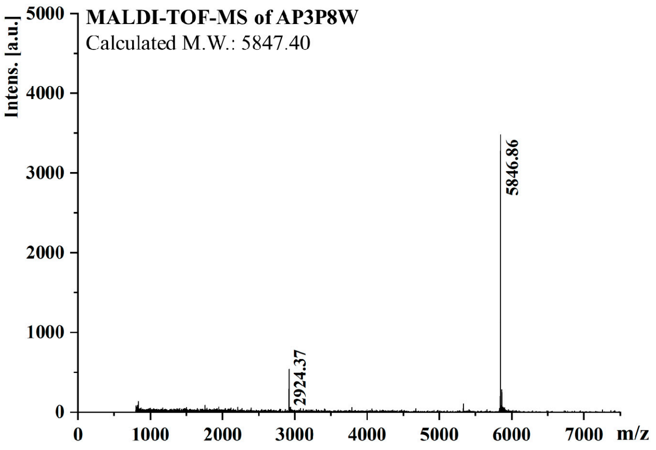

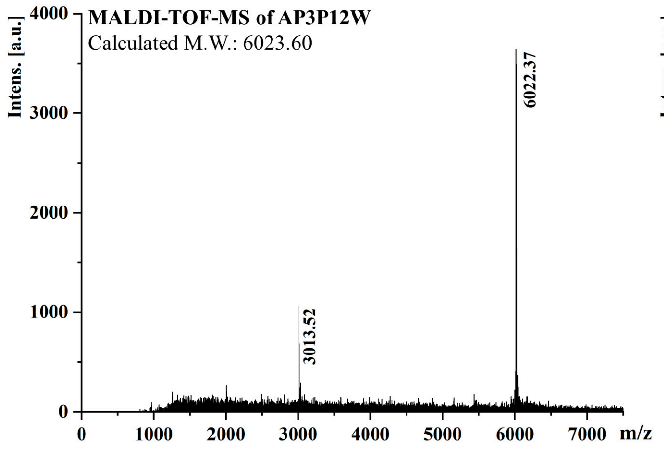

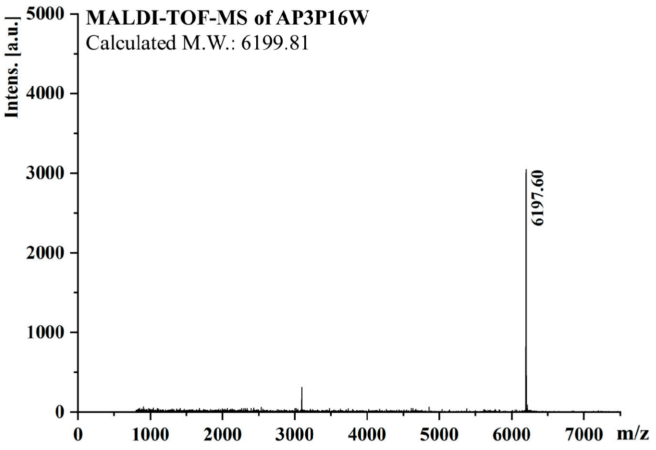

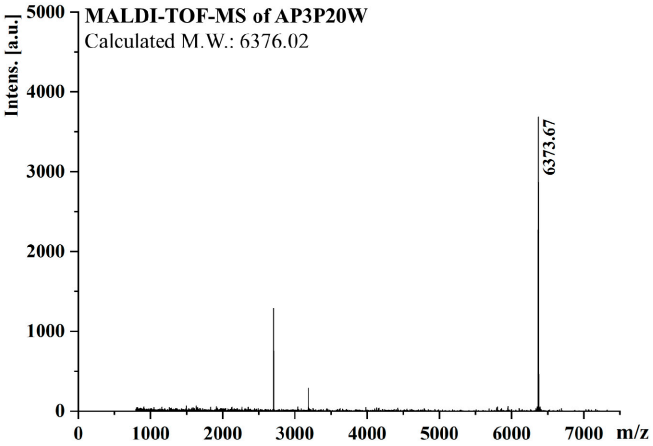
MALDI-TOF-MS of AP3P20W
Calculated M.W.: 6376.02
6373.67
Intens. [a.u.]
m/z

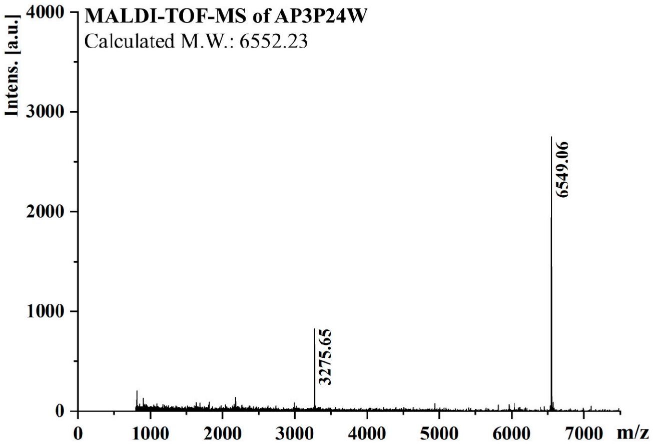
MALDI-TOF-MS of AP3P24W
Calculated M.W.: 6552.23
6549.06
3275.65
Intens. [a.u.]
m/z

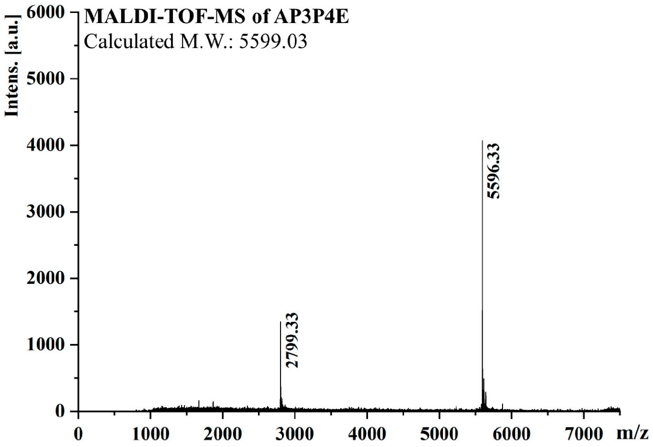
MALDI-TOF-MS of AP3P4E
Calculated M.W.: 5599.03
5596.33
2799.33
Intens. [a.u.]
m/z

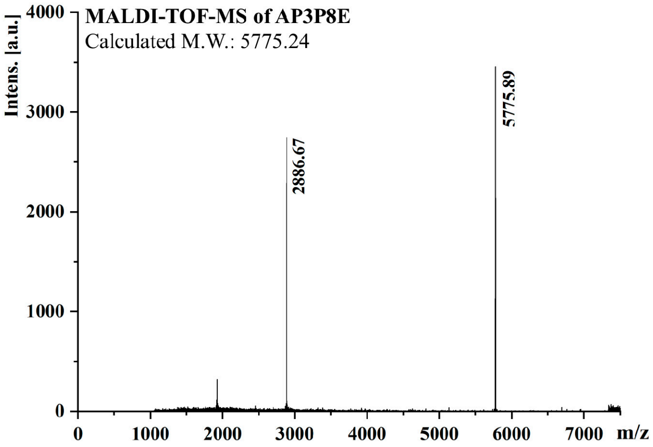
MALDI-TOF-MS of AP3P8E
Calculated M.W.: 5775.24
5775.89
2886.67
Intens. [a.u.]
m/z

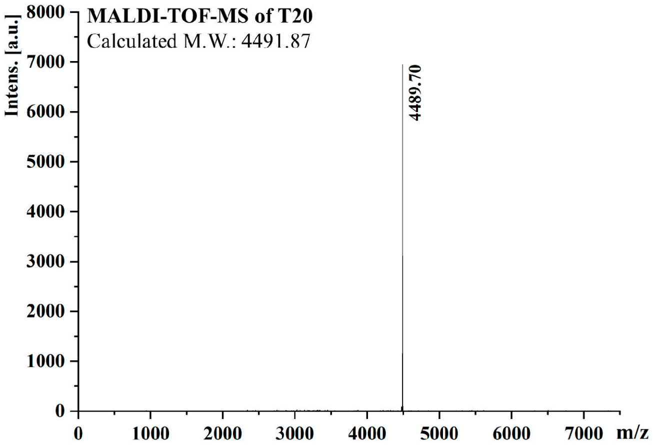
MALDI-TOF-MS of T20
Calculated M.W.: 4491.87
4489.70
Intens. [a.u.]
m/z

Analytical HPLC of AP3
[97% purity, t_R (Method A)= 12.4 min]

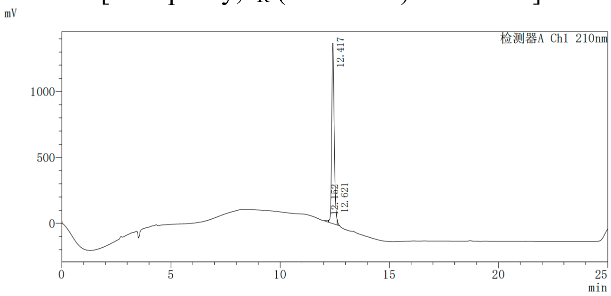

Analytical HPLC of AP3
[96% purity, t_R (Method B)= 14.9 min]

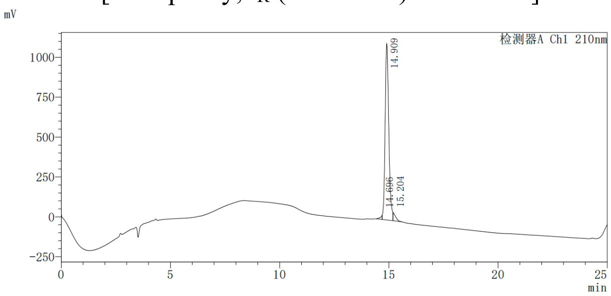

Analytical HPLC of AP3W
[99% purity, t_R (Method A)= 12.4 min]

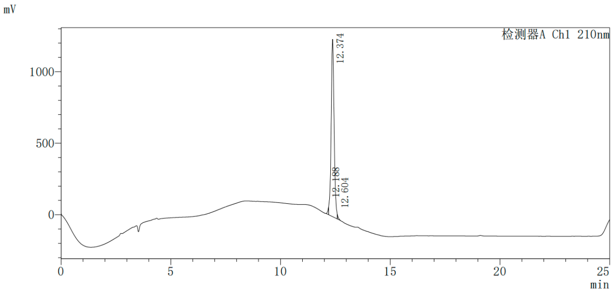

Analytical HPLC of AP3W
[96% purity, t_R (Method B)= 15.3 min]

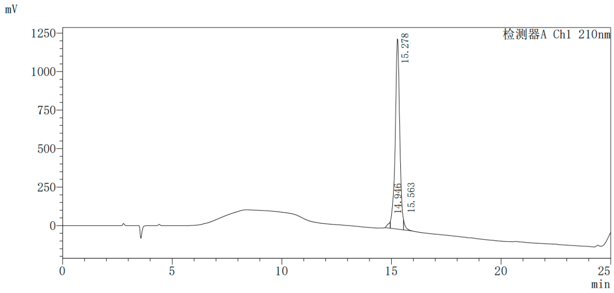

Analytical HPLC of AP3P4W
[99% purity, t_R (Method A)= 12.5 min]

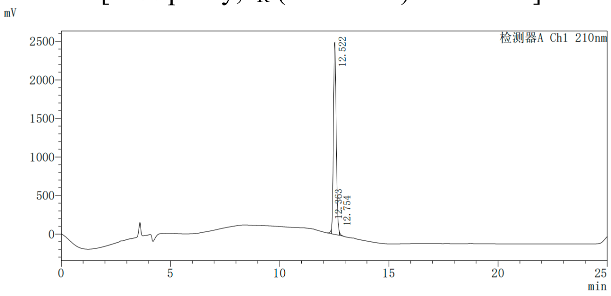

Analytical HPLC of AP3P4W
[99% purity, t_R (Method B)= 15.2 min]

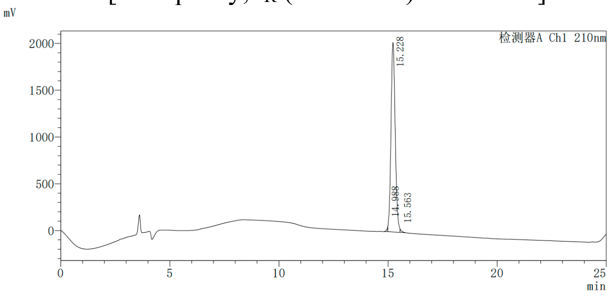

Analytical HPLC of AP3P8W
[99% purity, t_R (Method A)= 12.3 min]

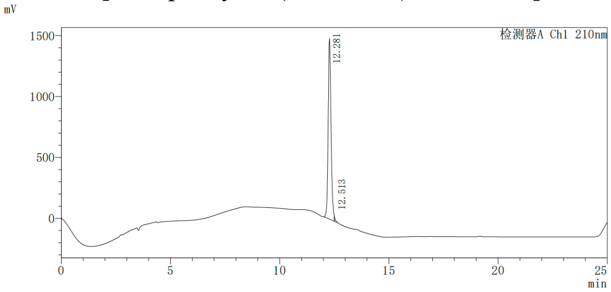

Analytical HPLC of AP3P8W
[98% purity, t_R (Method B)= 15.2 min]

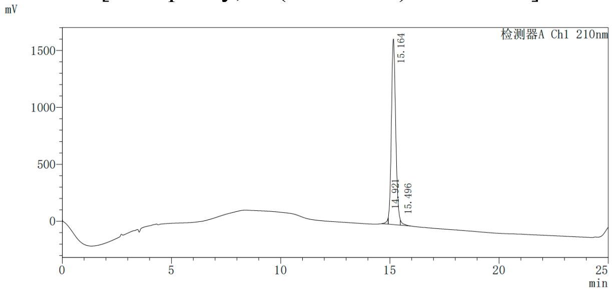

Analytical HPLC of AP3P12W
[99% purity, t_R (Method A)= 12.5 min]

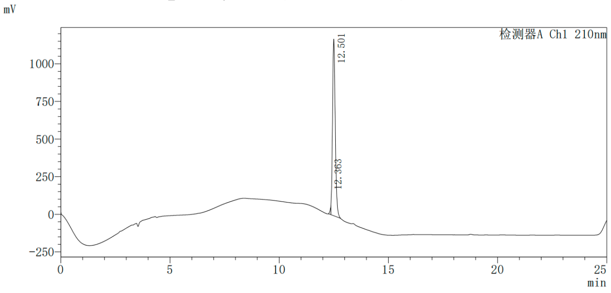

Analytical HPLC of AP3P12W
[98% purity, t_R (Method B)= 14.7 min]

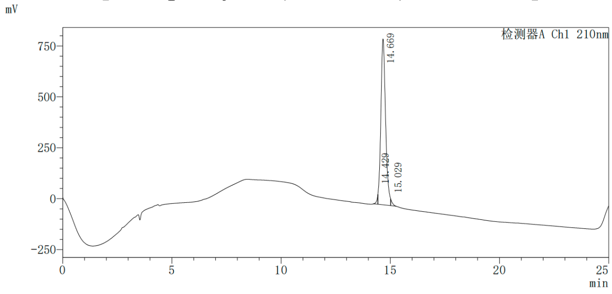

Analytical HPLC of AP3P16W
[99% purity, t_R (Method A)= 12.5 min]

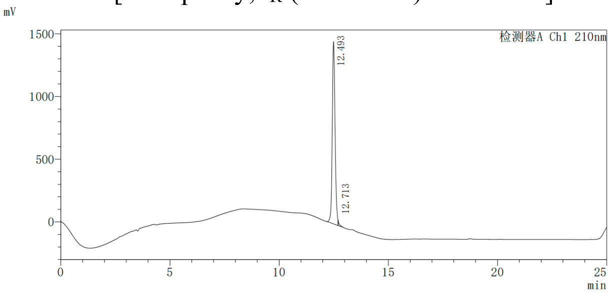

Analytical HPLC of AP3P16W
[98% purity, t_R (Method B)= 15.2 min]

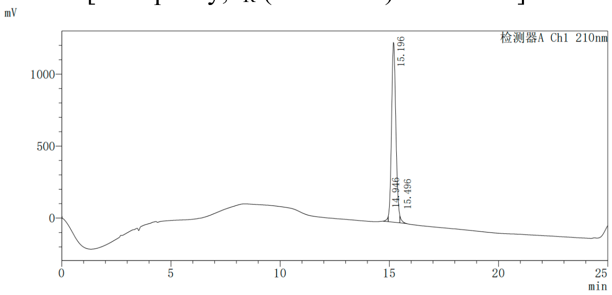

Analytical HPLC of AP3P20W
[99% purity, t_R (Method A)= 12.5 min]

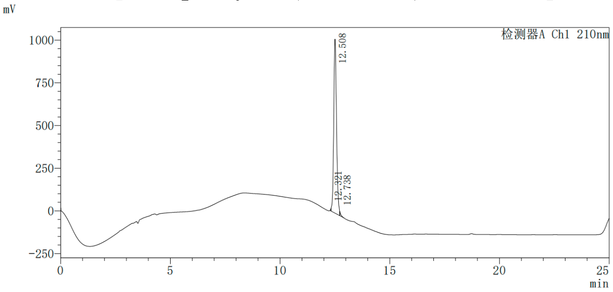

Analytical HPLC of AP3P20W
[99% purity, t_R (Method B)= 15.2 min]

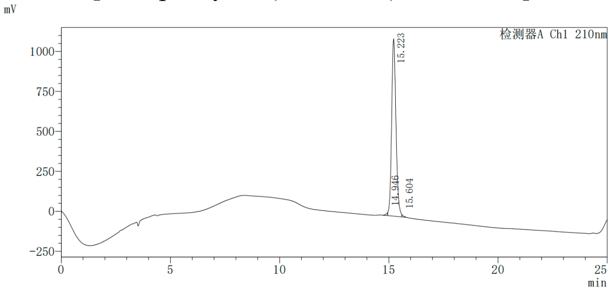

Analytical HPLC of AP3P24W
[99% purity, t_R (Method A)= 12.5 min]

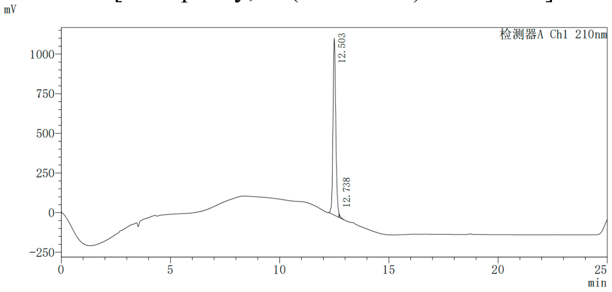

Analytical HPLC of AP3P24W
[98% purity, t_R (Method B)= 15.2 min]

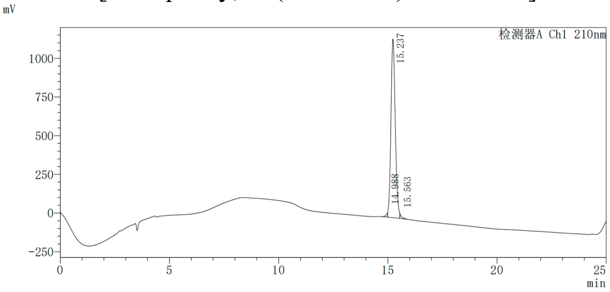

Analytical HPLC of AP3P4E
[99% purity, t_R (Method A)= 12.6 min]

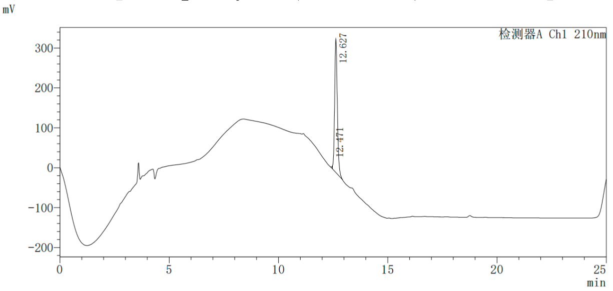

Analytical HPLC of AP3P4E
[97% purity, t_R (Method B)= 15.5 min]

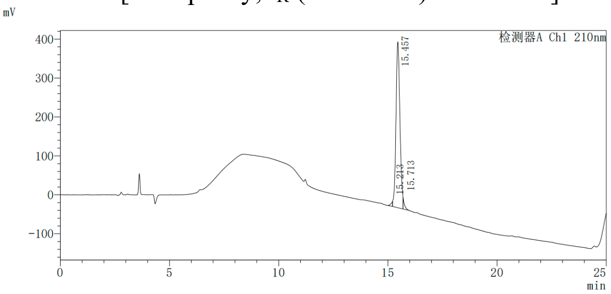

Analytical HPLC of AP3P8E
[99% purity, t_R (Method A)= 12.5 min]

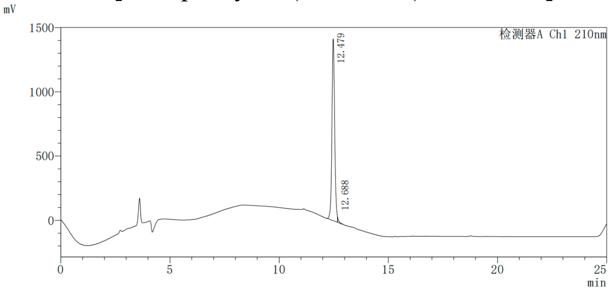

Analytical HPLC of AP3P8E
[98% purity, t_R (Method B)= 15.2 min]

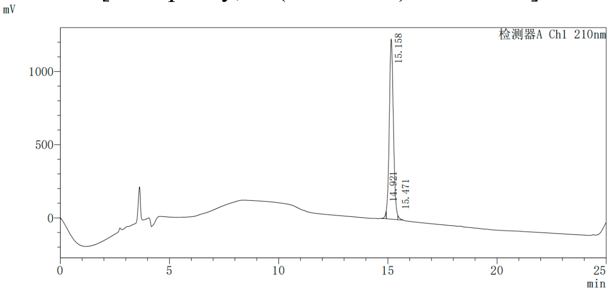

Analytical HPLC of T20

[98% purity, t_R (Method A)= 12.5 min]

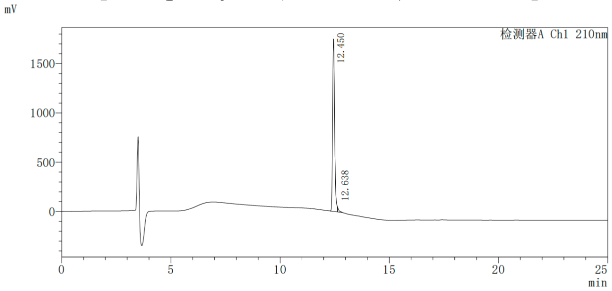

Analytical HPLC of T20

[96% purity, t_R (Method B)= 16.8 min]

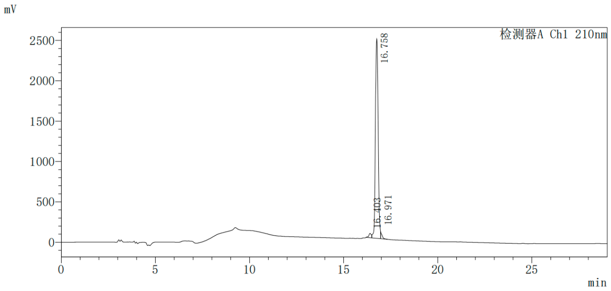

Supplement: Supplementary file 1 [file viruses-15-01038-s001.zip › viruses-2337661-supplementary.pdf]
